# Stomatitis.—Diarrhœa in Infants

**Published:** 1890-12

**Authors:** H. A. Hare

**Affiliations:** Clinical Professor of diseases of children, University of Pennsylvania, etc, etc.


ARTICLE II.

### STOMATITIS.—DIARRHŒA IN INFANTS.

BY H. A. HARE, M. D.,

Clinical Professor of diseases of children, University of Pennsylvania, etc, etc.

### STOMATITIS.

*Gentlemen:* The first case I show you to-day illustrates a disease which is frequently seen in children, but generally in those not so far advanced in age as is this boy. It is a case of ulcerative stomatitis, so called. Stomatitis is a very important disease, affecting, as it does, at one period of life, almost every child, producing at times very serious systemic disturbances, and occasioning considerable anxiety on the part of the parents. It may also at times produce reflex nervous trouble, such as a slight spasm. This affection has been divided into different classes by various authors, all of whom appear to confound more or less the various forms.

### CATARRHAL STOMATITIS.

According to the best authorities, the simplest form of stomatitis is the catarrhal form. This amounts to nothing more than a simple hyperemia of the entire mucous membrane of the mouth, which may extend to that of the pharynx, and perhaps down to œsophagus, or at times even through the entire alimentary canal. It corresponds to an acute cold in the head, so far as the pathological changes in the mucous membrane are concerned. Under these circumstances we are generally called to see the child because it has stopped nursing, or, if it be older, because it complains of a sensation of heat in the mouth, attended with a great deal of slobbering, and associated with crying and a constant moving of the hand to the mouth. Occasionally there is a slight febrile movement. Upon placing the finger in the mouth it is found to be hot to the touch and inflamed. This is probably due to the fact that there is an increased hyperemia, and consequently an increased local temperature. This is the simplest and mildest form of the disease. A little care devoted to the cleansing of the nipple, or regulating the diet, together with the use of some febrifuge mixture, such as the citrate of potassium and water, will relieve the child in a few hours, unless the attack be so severe as to bring on the aphthous form, or the mild ulcerative form.

### APHTHOUS STOMATITIS.

Aphthous stomatitis is really in one sense an ulcerative stomatitis, and yet it is not called so in the text-books. It is a stomatitis in which the catarrhal process has been so severe, particularly about the mucous follicles, that it has resulted in the death in small spots of the epithelium of the tongue and buccal mucous membrane. Small superficial ulcerations are formed, without any induration around their bases, and are not excavated—a form, practically, of a very mild ulcerative stomatitis. Aphthous stomatitis has been confounded by many writers with thrush. When a child comes to you with white, raised spots upon the tongue, that is thrush; if, instead, there are simple ulcerations upon the tongue, that is aphtha. In aphthous stomatitis, or "baby's sore mouth," as it is frequently called, the child loses its appetite, has a high fever, sooner or later develops diarrhœa or obstinate constipation, and may present severe nervous symptoms, twitchings of the muscles, and turning in of the thumbs. This condition requires careful treatment. It should be watched closely. A mixture may be prescribed containing:

| | | |
|---|---|---|
| R | Potass. chlorat. - - | ʒ ss-i. |
| | Tinct. myrrhæ - - - | gtt. v-x. |
| | Elixir. calisayæ - - | f ℥ iii. |

M. S. One teaspoonful every three or four hours.

As a rule children do not object to this mixture, since it is agreeable to the taste. Why do I give this? Because the chlorate of potassium is eliminated chiefly by the salivary glands, thereby bathing the mucous membrane with medicated saliva, and it is practically impossible to make a local application to the sore spots in young children. As a rule, marked relief speedily follows the use of such a prescription. One contra-indication only exists, and that is any acute renal inflammation, which, however, is a rare condition in childhood, unless it follows an acute disease. In such cases we must resort to other treatment, or use the chlorate in very small doses.

### ULCERATIVE STOMATITIS.

We now come to what is known as the true ulcerative stomatitis; and this is a disease which in severity greatly surpasses aphthous stomatitis. It is accompanied by the formation of pocketed ulcerations over the mucous membrane of the mouth, depressed, serpiginous, with crater-like edges and yellow bases, surrounded by induration, and often confluent, spreading over the lips and even down upon the chin in bad cases. There is a constant dribbling of saliva in all forms, wetting the skin of the chest. This is frequently followed by an eruption of eczema over the chest and abdomen. The nasal mucous membrane becomes involved in the process, and the nostrils are stopped up, resulting in breathing through the mouth. This causes the tongue to become as dry and scaly as it does in typhoid fever. Here the local treatment is identical with that in the other varieties. If the child is old enough, the ulcerations should be touched up with the stick of silver nitrate or a strong solution of the same salt. The stronger solutions hurt less than do the weak, and a solution containing from twenty to thirty grains to the ounce is the best for this purpose. It should be applied with a camel's hair brush, care being taken that the brush is not dripping wet. All that is necessary is to moisten the surface of the ulcers. At times a little cocaine may be used, but generally the burning of the cocaine is worse than that of the silver, and we thus use two medications instead of one. Fewer mixtures of various kinds are wanted. Particular attention should be paid to the bowels and kidneys. The urine is always found to be concentrated, and it is not only well to use the chlorate of potassium solution already named, but it is also wise to order Vichy or some other mineral water, which will act upon the liver and at the same time wash out of the kidneys the potassium products and lower the specific gravity of the urine.

### NOMA—CANCRUM ORIS.

We now advance to another form of ulceration of the mouth, known as cancrum oris or noma, which is practically not a stomatitis. We might say that this is an invariably fatal condition. Perforating ulcers appear upon the mucous membrane, going on to gangrene, causing sloughing away of the cheeks, so that the inferior maxilla is exposed. It is accompanied by great loss of flesh, wasting, high fever, cachexia and rapid death. It is a very rare condition in this country, and is probably due to some micro-organism not yet identified. We may use a supporting treatment and apply silver nitrate and other caustics locally; but death usually follows.

This boy has the ordinary mild stomatitis, a little too severe for aphtha, and too mild for the ulcerative form. In some cases of the aphthous and milder ulcerative varieties, and always in the severe forms, the systemic depression that is produced is simply appalling. Within twenty-four hours the child will sink into a complete collapse. Many English writers consequently believe that alcohol should always be used in these cases; and this is a matter it will be well to bear in mind.

You will find among nurses, and particularly monthly nurses, that they always assert that this disease is bound to "go through" the child. Practically this is so. I hardly ever see a case of stomatitis which is not followed by a catarrh of the bowels and stomach, and the patient is usually ill at least ten days. In such cases, as soon as the diarrhœa becomes excessive, some astringent mixture may be given. But do not check such a diarrhœa too rapidly or serious consequences may follow, and even convulsions in young infants. Paregoric or aromatic sulphuric acid are best for this purpose, their use being stopped as soon as the stools are reduced to two or three in the day.

### THRUSH.

I have passed over the condition known as thrush, which is often incorrectly called "baby's sore mouth." Thrush is an entirely different disease, due to the presence in the mouth of saccharomyces albicans. It is due to a fungus and differs from aphthous stomatitis in the fact that it grows from the surface like tufts of mould. Its size varies from that of a pin's head to that of a small finger nail. Thrush is found upon the tip and edges of the tongue as a rule. Practically it presents the same symptoms as does stomatitis: a hot mouth, dribbling, inability to take food on account of the severe pain, although the child is intensely hungry. This is one reason why collapse is so frequent in stomatitis. If the child is old enough to take a mouth-wash, give it a mild carbolized solution, say a one per cent. solution of the crystallized carbolic acid, washing the mouth every two or three hours. The chlorate of potassium is not as useful here as in stomatitis. The carbolic acid is more of an antiseptic and hence is more serviceable. Thrush rarely extends down through the alimentary canal. Some clinical authorities say they have found the fungus in the stomach and even in the small intestines; but this is very rare.

The boy I showed you with the ulcerations around the edge of each tooth has a condition somewhat different from the ordinary stomatitis, in the fact that the ulceration has extended to the gums. It is comparatively rare for this to occur. I do not know what he received from the dispensary. He came in on Saturday for the first time, and is better since using his mouth-wash. An excellent remedy to use as a wash is the sulpho-carbolate of sodium, five to ten grains to the ounce. This is perfectly innocuous, thus having an advantage over carbolic acid.

One word as to the diet in these cases. Parents always want to know about the diet. This is a very important matter. We cannot cure our patients unless it is attended to. Do not give anything which contains sugar. Cut down the sweetening of the bottle, or use instead a small amount of saccharine. For an older child, order broths, thin, non-greasy soups, and largely a milk diet, washing out the mouth with an antiseptic wash after each meal.

### SEROUS DIARRHŒA.

The baby I now show you is three months old, and has had a watery diarrhœa for the past two weeks attended with but little pain, the child crying only occasionally. This is generally the case with a watery diarrhœa. In mucous diarrhœa we nearly always have tenesmus and pain. The child has been fed upon condensed milk, one part to sixteen of water ever since its birth until last Saturday, when it was ordered cow's milk. Undoubtedly this attack the child is suffering from is due to the use of improper food. It was getting condensed milk instead of human or cow's milk. This is one class of cases in which we are apt to meet with thrush.

### MUCOUS DIARRHŒA.

The first case you see of mucous diarrhœa in children will puzzle you. It must be controlled; but how can this be done. The first thing is to remove the cause, and then to cure the condition produced by the cause. The mother now tells me that the baby has some green in its stools. This does not generally occur in serous diarrhœa. The presence of green usually indicates a mucous and not a serous diarrhœa. These green spinach stools of young children are always due to fermentation, and we rarely have fermentation when we have a serous diarrhœa. A small indigestible particle of food lying in the intestinal canal produces an irritation of the mucous membrane and consequent depression of vitality. A gradually spreading catarrh is produced with a mucous secretion, thick and tenacious, which coats the particles of food. These are then rolled along in the canal until finally the child has in its alimentary canal a decomposing piece of milk or meat covered with mucus which cannot be digested. Active inflammation is set up, and fermentation follows, accompanied with the growth of a fungus. These passages are nearly always acid, instead of being alkaline. Should you give the child an astringent mixture now, you will do it harm. Nature is trying to relieve the child by washing the irritating substance out. Something must be given to clear out the alimentary canal. The best thing is a dose of castor oil, a soothing purge taking five hours to act and facilitating the discharge of the immense amount of mucous thrown out in the alimentary canal. Then administer the astringent mixture. If the child has a puffy distended belly, indicating a chronic condition, follow the purge with tonics and alkalies. A mixture of bicarbonate of sodium and gentian is the best remedy for such cases. This primary purgative treatment will occasionally be objected to by the parents, and then it must be explained. If we are compelled to give an astringent, one of the best for a young baby is chalk mixture. This, however, is hard to swallow, and it may be better to give one half or one drop of aromatic sulphuric acid to a child of six months or one year, combined with syrup of ginger, elixir curacoa, or other adjuvant, to render it palatable. A mixture of bicarbonate of soda, forty-eight grains to a drachm, in six ounces of compound infusion of gentian is an excellent preparation, given in dessert-spoonful doses. If the compound tincture of gentian is used, the dose must be less. The only objection to this is its bitter taste; but this may be overcome by administering a peppermint cream drop before and after giving the dose. The condition of acidity of the alimentary canal is overcome by the alkali, while the compound tincture of gentian is a tonic, overcoming the depression which results from the primary irritation and inflammation.—*Med. and Surg. Reporter.*

